# Strength Training Versus Walking on the Fibromyalgia Impact: A Blinded Randomised Controlled Trial

**DOI:** 10.1002/msc.70186

**Published:** 2026-02-11

**Authors:** André Pontes‐Silva, Almir Vieira Dibai‐Filho, Thayná Soares de Melo, Leticia Menegalli‐Santos, Josimari Melo DeSantana, Marcelo Cardoso de Souza, Mariana Arias Avila

**Affiliations:** ^1^ Department of Physical Therapy Study Group on Chronic Pain (NEDoC) Laboratory of Research on Electrophysical Agents (LAREF) Postgraduate Program in Physical Therapy Universidade Federal de São Carlos (UFSCar) São Carlos São Paulo Brazil; ^2^ Department of Physical Education Postgraduate Program in Physical Education Universidade Federal do Maranhão São Luís Maranhão Brazil; ^3^ Department of Physical Therapy Laboratory of Research on Neuroscience (LAPENE) Graduate Program in Health Science Graduate Program in Physiological Science Universidade Federal de Sergipe Aracaju Sergipe Brazil; ^4^ Department of Physical Therapy Universidade Federal do Rio Grande do Norte Natal Rio Grande do Norte Brazil

**Keywords:** aerobic exercise, musculoskeletal system, pain, quality of life, rehabilitation

## Abstract

**Objective:**

To compare the effect of 24 sessions of progressive intensity strength training on the impact of fibromyalgia (primary outcome). Furthermore, we evaluated its effects on sleep, anxiety, depression, wind‐up mechanism, conditioned pain modulation, cutaneous sensory threshold, musculoskeletal performance, walking ability, perceived improvement, and treatment adherence (secondary outcomes).

**Design:**

A blinded randomised controlled trial.

**Setting:**

After blinded outcome assessments, 66 people were randomised and concealed and allocated to progressive (*n* = 22), constant (*n* = 22), or walking (*n* = 22) strength training groups.

**Participants:**

People with fibromyalgia.

**Interventions:**

In the progressive group, exercise intensity increased by 20% of maximum strength each month: 50% in the first month, 70% in the second month, and 90% in the third month. In the constant or walking exercise groups, moderate intensity was maintained at the end of the treatment. Each person received 24 individual exercise sessions (2x/week), with three months of exercise and three months of no exercise.

**Main Outcome Measure:**

Fibromyalgia impact.

**Results:**

Groups were similar at baseline. There were no significant between‐group differences in the primary outcome at any time point. In within‐group comparisons, we observed significant differences indicating that all three types of exercise reduced fibromyalgia symptoms; however, no variable achieved a minimal clinically important difference. In between‐group comparisons for the secondary outcomes, groups reported a positive perception of improvement, but most of each group did not adhere to treatment and/or did not answer about adherence after follow‐up without exercise.

**Conclusions:**

Twenty‐four sessions of progressive intensity strength training did not provide a greater reduction in the fibromyalgia impact than constant intensity or walking exercises.

**Trial Registration:**

Brazilian Registry of Clinical Trials (ReBEC): RBR‐9pbq9fg, date of registration: October 06, 2022.

**Protocol Publication:**

BMC Musculoskeletal Disorders: Doi – 10.1186/s12891‐023‐06952‐3 | Published: Volume 24, article number 816, October 14, 2023.

## 1 Introduction

Fibromyalgia is a chronic condition characterised by widespread pain and complex symptoms, including fatigue, mood disturbances, and functional symptoms (Pontes‐Silva, Dibai‐Filho, et al. 2023a). To date, non‐pharmacologic treatment recommendations that suggest physical exercise are based on systematic reviews that have examined different types of exercise, such as flexibility (Kim et al. 2019), mixed exercise (J Bidonde et al. 2019), strength training (Albuquerque et al. 2022; Vilarino et al. 2021), aquatic exercise (Bidonde et al. 2014), and walking (Bidonde et al. 2017). In terms of evidence, it is known that strength training is more effective than flexibility training in improving fibromyalgia symptoms (Busch et al. 2013). It is also known that walking is one of the most cited exercises in the literature for the treatment of fibromyalgia (Bidonde et al. 2017). However, studies are unclear about strategies for adapting exercise intensity to the biological adaptations of people with fibromyalgia are unclear, making it difficult to apply the findings clinically (Pontes‐Silva, Dibai‐Filho, et al. 2023a).

Current scientific evidence suggests that high‐intensity strength training is safe for people with fibromyalgia; however, there is a lack of guidance in the literature on methods of load progression to achieve such intensities (Busch et al. 2013; Da‐Silva et al. 2021). In this regard, there are two systematic reviews that examined variables related to strength training for people with fibromyalgia (Busch et al. 2013; Da‐Silva et al. 2021). The first review suggests that strength training should be performed at moderate or high intensity for people with fibromyalgia (Busch et al. 2013). The second review describes some planning parameters, such as, frequency of twice a week, intensity of 40%–80% of maximum strength, and volume of one to two sets of four to 12 movements for the relevant muscle groups: gastrocnemius, quadriceps, hamstrings, pectorals, latissimus dorsi, rhomboids, deltoids, biceps, and triceps (Da‐Silva et al. 2021).

However, previous studies using these parameters have produced controversial results, raising further doubts about their effects on the fibromyalgia impact (Andrade et al. 2018; Ernberg et al. 2018, 2016). Another weakness is that the comparison between‐studied groups did not show the progression of strength training intensity (Pontes‐Silva, Dibai‐Filho, et al. 2023a). In addition, studies that used strength training selected only one type of intensity in the research (progressive or constant), which increases the gap regarding the effects of each of the intensities on fibromyalgia and justifies the conduct of this study (Pontes‐Silva, Dibai‐Filho, et al. 2023a). Given this finding, the following question arises: does progressive intensity strength training promote a greater reduction in the levels of fibromyalgia impact? Our hypothesis is that progressive intensity strength training produces a greater reduction in fibromyalgia impact than other constant intensity exercises (e.g., walking or the same strength training programme).

Therefore, as a primary outcome, we aimed to compare the effect of 24 sessions of progressive intensity strength training (compared to constant intensities) on the fibromyalgia impact. Furthermore, as secondary outcomes, we evaluated its effects on sleep, anxiety, depression, wind‐up mechanism, conditioned pain modulation, cutaneous sensory threshold, musculoskeletal performance, walking ability, perceived improvement, and treatment adherence.

## 2 Methods

### 2.1 Trial Design and Ethical Aspects

A blinded randomised controlled trial was described according to the consolidated standards for reporting clinical trials (CONSORT) and the template for intervention description and replication (TIDieR). The research was conducted at the *Unidade Saúde Escola (USE) of the Federal University of São Carlos (UFSCar), Brazil,* between June 2022 and January 2025. All procedures of this study were previously approved by the Human Research Ethics Committee of the aforementioned institution (report number: 5.499.078).

Prior to the start of data collection, the study was also approved by the Brazilian Clinical Trials Registry (report number: *RBR‐9pbq9fg*). Subsequently, the research was publicised through social media (WhatsApp, Facebook, Instagram, Twitter), through the university's dissemination channels, and through flyers and posters in public health services in the city of São Carlos (SP), Brazil. In addition, the protocol of this clinical trial was published in the format of an open‐access scientific article, with all the details for public consultation (Pontes‐Silva, Dibai‐Filho, et al. 2023a).

### 2.2 Sample Size

We used Ene 3.0 and G*Power 3.1.9.7 software for sampling, considering an analysis of variance in the comparison among three independent groups in repeated measures: before and after 24 exercise sessions. We chose the fibromyalgia impact as the primary outcome variable. The calculation was based on detecting the minimal clinically important difference of 27 points between independent groups, standard deviation of 16.3 points, statistical power of 95%, significance of 5%, effect size of 0.41, and dropout rate of 15%. Thus, the sample should contain 21 people per group, for a total of 63 people with fibromyalgia (Pontes‐Silva, Dibai‐Filho, et al. 2023a).

### 2.3 Participants

People with fibromyalgia that were interested in participating in the study contacted the researchers via the WhatsApp provided. During the contact, they were instructed to read the digitally available consent form for signature and download, ask questions of the researcher in charge of the assessments, and carefully fill out a form on Google Forms that included the fibromyalgia screening, eligibility criteria, sample characterisation, and self‐report instruments for this study (Pontes‐Silva, Dibai‐Filho, et al. 2023a).

We recruited individuals ageing from 20 to 55 years to participate in the study by giving free, prior, and informed consent. Inclusion criteria were: diagnosis of fibromyalgia according to the 2016 recommendations (Wolfe et al. 2016). Exclusion criteria were: neurological conditions that interfered with the assessments, such as paralysis, significant sensory changes and level of consciousness/understanding; advanced joint disease; suspected thrombosis, heart disease and immediate postoperative period; pregnancy; alcohol and illicit substance abuse; and active cancer (Pontes‐Silva, Dibai‐Filho, et al. 2023a).

### 2.4 Randomisation, Allocation Concealment, and Blinding

The sample was randomised into three groups: group 1, which received progressive intensity strength training (experimental); group 2, which received constant intensity strength training (control A); and group 3, which received constant intensity walking (control B). The researcher responsible for recruitment, eligibility, and assessments was blinded to the interventions, as she did not know which group each person with fibromyalgia was assigned to (allocation concealment) (Pontes‐Silva, Dibai‐Filho, et al. 2023a).

After all, assessments, the bachelor of physical education (therapist, with seven years of experience), who was blinded to the assessments, handed out an opaque, sealed envelope containing 66 sequentially numbered pieces of paper describing one of the three groups. The person with fibromyalgia then drew a single piece of paper, without any influence from the researchers, and was thus randomly assigned. Finally, people with fibromyalgia were instructed not to share information about the assessments and interventions with anyone involved in the research (Pontes‐Silva, Dibai‐Filho, et al. 2023a).

### 2.5 Outcomes

The primary outcome of this study was the fibromyalgia impact, as this is the most commonly assessed self‐reported measure in people with fibromyalgia. Therefore, this study focused on comparing the effect of 24 sessions of progressive intensity strength training versus constant intensity (e.g., walking or the same strength training programme) on the primary outcome.

As a secondary outcome, this study focused on evaluating the effects of the intervention on other measures relevant to people with fibromyalgia, such as sleep quality, anxiety, depression, wind‐up mechanism, conditioned pain modulation, cutaneous sensory threshold, musculoskeletal performance through isokinetic dynamometry, walking ability, perception of improvement (weeks 6 and 12), and adherence to treatment through a 3‐month follow‐up without exercise (week 24).

### 2.6 Assessments

After voluntary and informed completion of the Google Forms, each person with fibromyalgia was individually invited to participate in the in‐person assessments, which included handgrip strength, sample characterisation, and other tests to obtain the secondary outcome variables.

### 2.7 Fibromyalgia Rapid Screening

We used the fibromyalgia rapid screening tool to screen for the disease (Perrot et al. 2010). This instrument was validated by Sousa et al. for the Brazilian population, with an adequate intraclass correlation coefficient and internal consistency. It is a self‐administered instrument consisting of six items with the response options ‘yes’ or ‘no’, with a cut‐off point of five points, indicating that people scoring five or six points are more likely to have fibromyalgia (De Sousa et al. 2022).

### 2.8 Fibromyalgia Impact

We assessed the fibromyalgia impact using the revised fibromyalgia impact questionnaire, which was validated for the Brazilian population by Lupi et al. (2017) and has adequate reliability and internal consistency. The instrument was used for the first‐, sixth‐, twelfth‐, and twenty‐fourth‐week assessments. The questionnaire contains 21 items assessing the function, global impact, and symptoms of fibromyalgia. All questions relate to experiences in the past 7 days and are presented on a numerical rating scale from zero to 10 points. The score from zero to one hundred points is obtained by adding the three normalised domain scores. The lower the score, the lower the fibromyalgia impact. A difference of 27 points in the total score was considered a minimal clinically important difference between independent groups (Surendran and Mithun 2018).

### 2.9 Sleep

Sleep quality was assessed using the Pittsburgh sleep quality index. This instrument is reliable (Passos et al. 2017) and was adapted for Brazilians by Bertolazi et al. It assesses seven sleep components: subjective sleep quality, sleep latency, sleep duration, habitual sleep efficiency, sleep disturbances, use of sleep medications, and daytime dysfunction. Scores ranged from zero to three for each component, with a maximum score of 21. Scores above five indicate poor sleep quality (Bertolazi et al. 2011).

### 2.10 Anxiety and Depression

We assessed anxiety and depression using the hospital anxiety and depression scale, validated for Brazilians by Marcolino et al. This scale consists of 14 items divided into two domains: anxiety and depression, each with seven items. The items have a Likert scale with four numerical values, resulting in total scores ranging from zero to 21 for the two domains. The cut‐off points indicating moderate to severe symptoms are equal to or greater than eight for anxiety and equal to or greater than nine for depression (Marcolino et al. 2007).

### 2.11 Pain

Pain was assessed with different instruments and tests. Pain intensity was assessed using the numerical pain rating scale, validated for Portuguese by Ferreira‐Valente et al. It has a sequence of numbers between zero and 10. This instrument was used in the temporal summation (wind‐up mechanism) and conditioned pain modulation tests (Ferreira‐Valente et al. 2011). A two‐point reduction in pain intensity is considered a minimal clinically important difference (Farrar et al. 2001).

**FIGURE 1:**
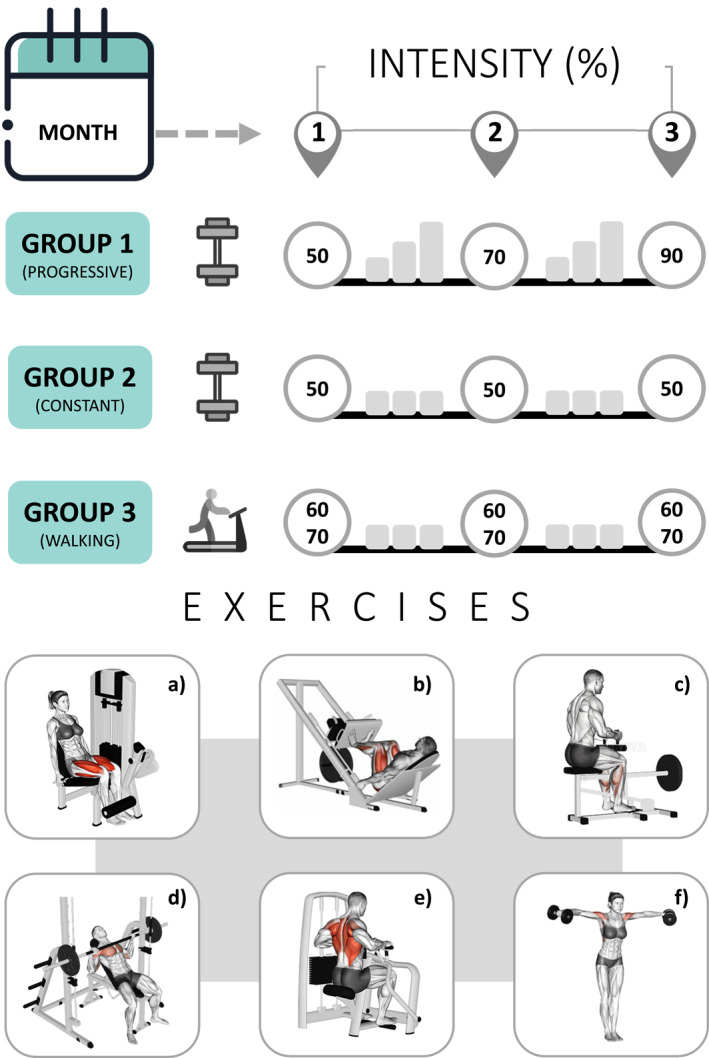
Exercises and their intensities throughout the treatment period.

We evaluated the winding mechanism by means of a temporal summation test using a digital pressure algometer (ITO, Tokyo, Japan), the reliability of which has already been demonstrated. The test verifies the progressive and frequency‐dependent facilitation of neuron's responses observed during the application of repetitive or constant intensity stimuli. A pressure of 2.5 kg was applied to the anterior surface of the right forearm of the person with fibromyalgia, 7.5 cm from the distal wrist crease, and maintained for 30 seconds. During this time, the person with fibromyalgia self‐reported the intensity of pain perceived at the first, tenth, twentieth, and 30th seconds of stimulus application (Figure 1) (Pontes‐Silva, Dibai‐Filho, et al. 2023a).

We evaluated conditioned pain modulation, which consists of reducing pain in response to another painful stimulus (conditioned stimulus). The test was divided into four steps: first, we measured the pressure‐pain threshold using the same positioning as in the temporal summation test to stimulate intensity four pain. Second, we applied ischaemic compression of 250 mmHg to the left arm using an analogue sphygmomanometer to stimulate intensity five pains. Third, we repeated the first phase of the test, but during the conditioned stimulus. Fourth, 5 minutes after the conditioned stimulus was removed, we repeated the first phase of the test (Figure 1) (Pontes‐Silva, Dibai‐Filho, et al. 2023a).

**FIGURE 2:**
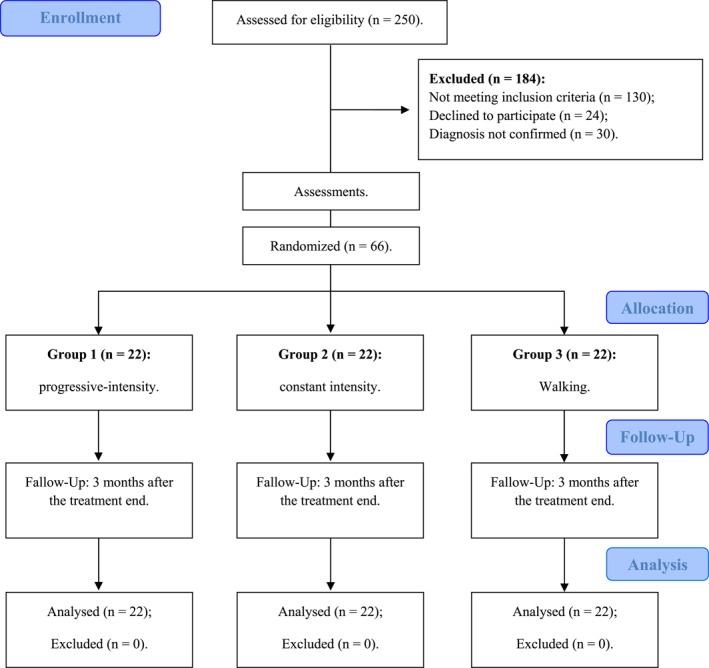
Flowchart.

We assessed the cutaneous sensory threshold using a set of von Frey filaments in the trapezius, supraspinatus, and sternocleidomastoid muscles. The person with fibromyalgia was blindfolded during the test. In order to increase the‐force, each filament was positioned perpendicular to the skin and gently squeezed to its initial bend and then removed. The first filament where the person reported feeling touch was considered the cutaneous sensation threshold (Figure 2) (Pontes‐Silva, Dibai‐Filho, et al. 2023a).

### 2.12 Musculoskeletal Performance: Isokinetic Dynamometry

We used a Biodex Medical Systems 3 (Shirley, New York, United States) for this evaluation. The person with fibromyalgia was positioned with the hip angle at 100° and the trunk, pelvis and thigh were stabilised with straps. The axis of rotation of the dynamometer was aligned with the axis of the knee, at the level of the lateral epicondyle of the femur, fixed to the distal part of the leg, 5 cm above the medial malleolus. Correction for the effect of gravity was calculated using the limb flexed at 60° (Pontes‐Silva, Dibai‐Filho, et al. 2023a).

Prior to the isokinetic assessment, we performed a familiarisation series of five submaximal isokinetic contractions. Three minutes after familiarisation, participants performed 10 concentric isokinetic knee extension contractions ranging from 90° to 15° with a total amplitude of 75° at an angular velocity of 60°/second. During all contractions performed on the right lower limb, verbal encouragement and visual feedback from the device were provided in an attempt to achieve maximum voluntary effort (Pontes‐Silva, Dibai‐Filho, et al. 2023a).

### 2.13 Walking Ability

We assessed walking ability using the six‐minute walk test, which has been shown to be reliable (Pankoff et al. 2000). We instructed the person with fibromyalgia to walk as fast as possible for the 6 minutes and to stop the test if they felt uncomfortable. Before and after the session, we monitored blood pressure, heart rate, oxygen saturation, and respiratory rate. The level of exertion during the test was monitored using the Borg scale. An increase of one hundred and 56 m in the distance walked is considered a minimal clinically important difference in fibromyalgia (Kaleth et al. 2016).

### 2.14 Muscle Strength for Maximum Dynamic Load When Exercising

Maximum muscular strength for the exercise load in strength training groups was determined using the one‐repetition maximum test. The results of this test, in addition to being reliable (Grgic et al. 2020), are widely used to identify the amount of kg based on the percentage of strength observed. The person with fibromyalgia performed 10 repetitions of the movement without load to warm up the musculoskeletal system and to understand the technique. After 1 minute of rest, three to five maximum repetitions were performed, as observed by the evaluator and self‐reported by the subject. After 3 minutes of rest, the one‐repetition maximum test was performed (Ribeiro et al. 2018).

The load used in the one‐repetition maximum test was determined by the maximum number of repetitions observed by the evaluator and reported by the person with fibromyalgia. The number of maximum repetitions was entered into the mathematical equation proposed by Brzycki, in which one maximum repetition is equal to the submaximal load in kg divided by (1.0278–0.0278 × number of repetitions performed). The test was used to adjust the intensity of strength training in the first, fourth and eighth weeks (Pontes‐Silva, Dibai‐Filho, et al. 2023a).

### 2.15 Global Perceived Effect Related to Treatment

We assessed self‐reported global perceived effect of treatment using the global perceived effect, an instrument validated for the Brazilian population. This is an eleven‐point descriptive scale in which the individual's perception is classified according to their score at a given time. Thus, the person with fibromyalgia reported their perception of improvement in relation to the treatment using a score ranging from −5 to +5 and classified as: much worse, no change or fully recovered. A change of three points is considered a minimal clinically important difference (Costa et al. 2008).

### 2.16 Intervention

All groups received a 45‐min pain education session prior to the exercise programme, and all exercise sessions took place individually in a private room. The person with fibromyalgia received 24 exercise sessions, with two forty‐minute sessions per week, and a ten‐minute rest period after each session. The person with fibromyalgia was instructed to stop the intervention at any time without having to give a reason. In addition, blood pressure, heart rate, peripheral oxygen saturation, intensity of widespread pain, and subjective perception of exertion were monitored before and after the session. Additionally, throughout the treatment, the researcher responsible for the intervention maintained weekly contact via WhatsApp with each person with fibromyalgia, twice a week (Pontes‐Silva, Dibai‐Filho, et al. 2023a).

### 2.17 Group 1: Progressive Intensity Strength Training

The person with fibromyalgia performed a four‐minute global warm‐up divided into four unloaded exercises: seated calf raises, lateral raise with dumbbells, leg press, and incline bench press. This was followed by strength training at moderate intensity (50% of maximum strength), which involved nine muscle groups: glutes, quadriceps, hamstrings, biceps brachii, triceps brachii, pectoralis major, calf, deltoid, and latissimus dorsi. In this protocol, six exercises were used, divided between the upper and lower limbs: (1) leg extension, (2) leg press, (3) seated machine calf raise, (4) incline bench press, (5) seated machine row, and (6) dumbbell lateral raise (Pontes‐Silva, Dibai‐Filho, et al. 2023a).

Moderate intensity (50% of maximum strength) was previously determined by a one‐repetition maximum test in each of the six proposed exercises (Figure 1). Muscle strength was individually reassessed every 4 weeks and the intensity of each exercise was increased by 20% of the observed value in kg, that is, 50% in the first month, 70% in the second month and 90% in the third month (Pontes‐Silva, Dibai‐Filho, et al. 2023a).

The intensity of the exercises (kg), rest between sets, number of sets, repetitions, time under tension and frequency of strength training were planned and supervised by a bachelor in physical education with experience in chronic pain. Twice a week for 3 months, the person with fibromyalgia performed three sets of each exercise, with 10 repetitions, 40 seconds of muscle tension, full range of motion, inhalation in the eccentric phase, and 60 seconds of rest between sets (Pontes‐Silva, Dibai‐Filho, et al. 2023a).

To maintain the same volume of work in strength training, add 20 seconds of rest between sets each month according to the progression of intensity. Namely, 60 seconds in the first month, 80 seconds in the second month, and one hundred seconds in the third month, since higher intensities require longer rest periods to maintain the same volume of work (number of sets, repetitions, and time under tension) (Pontes‐Silva, Lopes, et al. 2023b).

### 2.18 Group 2: Constant Intensity Strength Training

Moderate intensity (50% of maximum strength) was previously determined by testing one maximum repetition of each of the six suggested exercises (Figure 1). Muscle strength was also individually reassessed every 4 weeks. However, in this group, the intensity of each exercise was maintained at 50% of maximum strength until the end of treatment. Therefore, a constant intensity and 60 second of rest between sets were maintained from the first to the third month. The other procedures were exactly the same (Pontes‐Silva, Dibai‐Filho, et al. 2023a).

### 2.19 Group 3: Constant Intensity Walking

The person with fibromyalgia walked on the treadmill for 40 minutes at a moderate intensity, determined by the speed corresponding to a rate of 60%–70% of the maximum heart rate monitored by a portable oximeter (Figure 1). We estimated the maximum heart rate by age in years, taking into account the standard error of 10 heartbeats according to Garber et al. (2011). Therefore, if the person with fibromyalgia exceeded 60%–70% of the maximum heart rate, the treadmill speed was slowly reduced until the heart rate reached the suggested rate of 60%–70% (Pontes‐Silva, Dibai‐Filho, et al. 2023a).

### 2.20 Statistical Analysis

Statistical analyses were performed according to the intention‐to‐treat principle (Nagel et al. 2020). Numerical comparisons were performed using linear mixed models, taking into account the interaction between time × group factors, with Bonferroni adjustment for multiple comparisons. Categorical comparisons were performed using chi‐squared and Fisher's exact tests. For significant differences between means, we calculated the effect size using Cohen's *d*, with the following classifications: small (0.2), medium (0.5), and large (0.8) (Pontes‐Silva, Dibai‐Filho, et al. 2023a). Thus, data were presented as mean, standard deviation, adjusted difference between means, and 95% confidence interval (95% CI). We used a 5% significance level in all analyses, which were performed using SPSS software, version 17 (Chicago, IL, United States).

## 3 Results

After the study was announced, 250 people completed the form expressing interest in participating (Figure 2). Of these, 184 were not enroled because they did not meet the inclusion criteria. However, these people received a pain education booklet. The final sample of 66 people with fibromyalgia was randomised and concealed into three groups: progressive (*n* = 22), constant (*n* = 22), and walking (*n* = 22). Table 1 describes the characteristics' sample and dropout rates in each group. Non‐significant differences were observed in all comparisons (*p* ≥ 0.05).

**TABLE 1:** Sample characterisation.

Initial assessment variables	Progressive (*n* = 22)	Constant (*n* = 22)	Walking (*n* = 22)
Age (years)	44.77 (7.70)	42.31 (8.99)	42.95 (9.60)
Body mass (kg)	78.78 (15.06)	81.19 (18.46)	75.43 (14.73)
Stature (m)	1.63 (0.06)	1.61 (0.07)	1.64 (0.07)
Body mass index (m/kg^2^)	29.68 (5.74)	30.98 (6.46)	27.96 (5.60)
Fibromyalgia rapid screening (0–6)	5.59 (0.66)	5.36 (0.90)	5.40 (1.33)
Sex (female)	21 (95.4%)	21 (95.4%)	20 (90.9%)
Medication usage (yes)	15 (68.1%)	16 (72.7%)	17 (77.2%)
Ethnicity (self‐reported)			
White	18 (81.8%)	17 (77.2%)	13 (59.1%)
Brown	3 (13.6%)	4 (18.1%)	9 (40.9%)
Black	1 (4.5%)	1 (4.5%)	0
Dropout rate (*n* = 19/66)	5 (22.7%)	9 (40.9%)	5 (22.7%)

*Note:* Mean (standard deviation) or number (%). Insignificant difference observed through linear mixed models or chi‐square (*p* ≥ 0.05).

Table 2 shows the descriptive values and between‐group comparisons for the primary outcome at each study time point: group versus group at baseline and at weeks 6, 12, and 24 (follow‐up). Although we observed a significant difference (*p* ≤ 0.05) between the progressive and walking groups at baseline in the impact domain, the difference (4.90 [95% CI: 0.94; 8.86]) has a medium effect size (*d* = 0.57). The primary outcome and the a priori sampling were planned using the sum of all domains of the Revised Fibromyalgia Impact Questionnaire (total score: 0–100). In this questionnaire, all groups were similar (*p* ≥ 0.05), and none of them reached the minimal clinically important difference of 27 points.

**TABLE 2:** Descriptive values and between‐group comparisons at all study time points (group vs. group). Revised fibromyalgia impact questionnaire (primary outcome).

Primary outcome (descriptive)	Progressive (*n* = 22)	Constant (*n* = 22)	Walking (*n* = 22)
	Mean (95% confidence interval)		
Function (0–30)			
Baseline	17.01 (13.72, 20.30)	17.90 (14.61, 21.20)	21.04 (17.75, 24.33)
6th week	12.91 (10.11, 15.71)	14.06 (11.26, 16.86)	15.08 (12.27, 17.88)
12th week	11.19 (8.28, 14.09)	13.09 (10.18, 16)	14.84 (11.93, 17.75)
Follow‐up (24th week)	12.72 (10.26, 15.17)	11.46 (9.01, 13.92)	12.74 (10.29, 15.20)
Impact (0–20)			
Baseline	11.50 (9.22, 13.77)	13.09 (10.81, 15.36)	16.40 (14.13, 18.68)
6th week	8.30 (6.04, 10.57)	9.85 (7.58, 12.11)	10.19 (7.93, 12.45)
12th week	8.06 (5.71, 10.42)	8.84 (6.49, 11.20)	10.97 (8.62, 13.33)
Follow‐up (24th week)	9.53 (7.64, 11.42)	8.19 (6.30, 10.07)	9.13 (7.25, 11.02)
Symptoms (0–50)			
Baseline	34.52 (30.87, 38.17)	36.47 (32.82, 40.12)	39.86 (36.21, 43.51)
6th week	28.08 (24.27, 31.89)	29.69 (25.88, 33.50)	29.10 (25.29, 32.91)
12th week	26.89 (23.13, 30.65)	29.56 (25.81, 33.32)	29.71 (25.95, 33.46)
Follow‐up (24th week)	30.45 (26.59, 34.31)	28.77 (24.91, 32.63)	30.61 (26.75, 34.47)
Total (0–100)			
Baseline	63.03 (54.72, 71.34)	67.47 (59.16, 75.78)	77.31 (69, 85.62)
6th week	49.30 (41.26, 57.34)	53.61 (45.57, 61.64)	54.37 (46.34, 62.41)
12th week	46.15 (37.58, 54.72)	51.51 (42.94, 60.08)	55.53 (46.96, 64.09)
Follow‐up (24th week)	52.71 (45.14, 60.27)	48.43 (40.86, 55.99)	52.49 (44.93, 60.06)

**Between‐group comparisons**	**Progressive—Constant**	**Progressive—Walking**	**Constant—Walking**
	**Estimated mean difference (95% confidence interval)**		
Function (0–30)			
Baseline	0.89 (−4.83, 6.62)	4.03 (−1.69, 9.75)	3.13 (−2.59, 8.86)
6th week	1.15 (−3.72, 6.03)	2.16 (−2.70, 7.04)	1.01 (−3.86, 5.89)
12th week	1.90 (−3.15, 6.96)	3.65 (−1.40, 8.71)	1.75 (−3.31, 6.81)
Follow‐up (24th week)	−1.25 (−5.52, 3.01)	0.02 (−4.24, 4.29)	1.27 (−2.99, 5.54)
Impact (0–20)			
Baseline	1.59 (−2.36, 5.55)	4.90 (0.94, 8.86)*	3.31 (−0.64, 7.27)
6th week	1.54 (−2.39, 5.47)	1.88 (−2.05, 5.82)	0.34 (−3.59, 4.27)
12th week	0.78 (−3.31, 4.88)	2.90 (−1.19, 7.01)	2.12 (−1.97, 6.22)
Follow‐up (24th week)	−1.34 (−4.62, 1.94)	−0.39 (−3.68, 2.88)	0.94 (−2.33, 4.23)
Symptoms (0–50)			
Baseline	1.95 (−4.39, 8.30)	5.34 (−1, 11.69)	3.38 (−2.96, 9.73)
6th week	1.61 (−5.02, 8.24)	1.02 (−5.61, 7.66)	−0.58 (−7.22, 6.05)
12th week	2.67 (−3.86, 9.21)	2.81 (−3.72, 9.36)	0.14 (−6.40, 6.68)
Follow‐up (24th week)	−1.68 (−8.39, 5.03)	0.15 (−6.55, 6.87)	1.84 (−4.87, 8.55)
Total (0–100)			
Baseline	4.43 (−10.02, 18.90)	14.28 (−0.18, 28.74)	9.84 (−4.62, 24.30)
6th week	4.30 (−9.68, 18.29)	5.07 (−8.91, 19.06)	0.76 (−13.22, 14.75)
12th week	5.36 (−9.55, 20.27)	9.37 (−5.53, 24.29)	4.01 (−10.89, 18.93)
Follow‐up (24th week)	−4.28 (−17.44, 8.88)	−0.21 (−13.38, 12.95)	4.06 (−9.10, 17.23)

*Note:* Baseline: Sample characteristics before treatment. 6th week: Sample characteristics after 12 exercise sessions. 12th week: Sample characteristics after 24 exercise sessions. Follow‐up: Sample characteristics after 3 months without exercise, that is, at the 24th week in relation to the baseline.

*Significant difference observed through linear mixed models (*p* ≤ 0.05).

Table 3 shows the delta comparisons between groups for the primary outcome at all study time points (time–time vs. group–group). We observed a significant difference (*p* ≤ 0.05) between the progressive and walking groups at both baseline (−5.30 [95% CI: −9.58; −1.03], *d* = 0.94]) and week 12 follow‐up (−3.30 [95% CI: −6.59; −0.01], *d* = 0.72). These results indicate that constant‐intensity walking produced greater reductions in impact domain scores on the Revised Fibromyalgia Impact Questionnaire than progressive‐intensity strength training.

**TABLE 3:** Between‐group delta comparisons: time–time versus group–group. Revised fibromyalgia impact questionnaire (primary outcome).

Primary outcome (Δ)	Progressive–Constant	Progressive–Walking	Constant–Walking
	Estimated mean difference (95% confidence interval)		
Function (0–30)			
Baseline–6th week	0.25 (−5.14, 5.66)	−1.86 (−7.26, 3.54)	−2.12 (−7.52, 3.28)
Baseline–12th week	1.00 (−5.52, 7.54)	−0.37 (−6.91, 6.15)	−1.38 (−7.92, 5.15)
Baseline–Follow–up	−2.14 (−8.14, 3.85)	−4.00 (−10.00, 1.99)	−1.85 (−7.85, 4.14)
6th week–12th week	0.74 (−3.63, 5.13)	1.48 (−2.90, 5.86)	0.73 (−3.64, 5.12)
6th week–Follow–up	−2.40 (−6.36, 1.54)	−2.14 (−6.10, 1.81)	0.26 (−3.69, 4.22)
12th week–Follow–up	−3.15 (−7.69, 1.37)	−3.62 (−8.16, 0.90)	−0.47 (−5.00, 4.06)
Impact (0–20)			
Baseline–6th week	−0.05 (−4.09, 3.99)	−3.02 (−7.06, 1.01)	−2.97 (−7.01, 1.06)
Baseline–12th week	−0.80 (−5.35, 3.73)	−2.00 (−6.54, 2.54)	−1.19 (−5.73, 3.35)
Baseline–Follow–up	−2.93 (−7.20, 1.34)	−5.30 (−9.58, −1.03)*	−2.37 (−6.64, 1.90)
6th week–12th week	−0.76 (−4.34, 2.82)	1.02 (−2.56, 4.61)	1.78 (−1.80, 5.37)
6th week–Follow–up	−2.88 (−6.04, 0.27)	−2.28 (−5.44, 0.88)	0.60 (−2.55, 3.76)
12th week–Follow–up	−2.12 (−5.41, 1.17)	−3.30 (−6.59, −0.01)*	−1.18 (−4.47, 2.11)
Symptoms (0–50)			
Baseline–6th week	−0.34 (−7.24, 6.55)	−4.32 (−11.21, 2.57)	−3.97 (−10.87, 2.92)
Baseline–12th week	0.72 (−7.02, 8.46)	−2.52 (−10.26, 5.22)	−3.24 (−10.98, 4.49)
Baseline–Follow–up	−3.63 (−11.96, 4.69)	−5.18 (−13.50, 3.14)	−1.54 (−9.87, 6.78)
6th week–12th week	1.06 (−4.78, 6.91)	1.79 (−4.05, 7.64)	0.73 (−5.12, 6.58)
6th week–Follow–up	−3.29 (−9.28, 2.70)	−0.86 (−6.85, 5.13)	2.43 (−3.56, 8.42)
12th week–Follow–up	−4.35 (−10.12, 1.40)	−2.65 (−8.42, 3.10)	1.70 (−4.06, 7.46)
Total (0–100)			
Baseline–6th week	−0.13 (−14.58, 14.31)	−9.20 (−23.65, 5.24)	−9.07 (−23.52, 5.37)
Baseline–12th week	0.92 (−16.59, 18.43)	−4.90 (−22.41, 12.61)	−5.82 (−23.33, 11.69)
Baseline–Follow–up	−8.71 (−25.79, 8.35)	−14.49 (−31.57, 2.58)	−5.77 (−22.85, 11.30)
6th week–12th week	1.05 (−11.27, 13.38)	4.30 (−8.02, 16.63)	3.25 (−9.08, 15.58)
6th week–Follow–up	−8.58 (−19.72, 2.55)	−5.28 (−16.42, 5.85)	3.29 (−7.83, 14.43)
12th week–Follow–up	−9.64 (−21.90, 2.62)	−9.59 (−21.85, 2.67)	0.04 (−12.21, 12.31)

*Note:* Baseline: Sample characteristics before treatment. 6th week: Sample characteristics after 12 exercise sessions. 12th week: Sample characteristics after 24 exercise sessions. Follow‐up: Sample characteristics after 3 months without exercise, that is, at the 24th week in relation to the baseline.

*Significant difference observed through linear mixed models (*p* ≤ 0.05).

Table 4 shows the within‐group comparisons for the primary outcome at all study time points. Specifically, the comparisons are between time points: baseline–week 6, baseline–week 12, baseline–follow‐up, week 6‐weeks 12, week 6–follow‐up, and week 12–follow‐up. Considering all the observations in Table 4, we found a significant difference (*p* ≤ 0.05, *d* > 0.5) in the total score of the Revised Fibromyalgia Impact Questionnaire at baseline and week 6 and at baseline and week 12. This indicates that the three types of exercises reduced global fibromyalgia symptoms. However, none of the exercises reached the minimal clinically important difference of 27 points.

**TABLE 4:** Primary outcome within‐group comparisons (time vs. time). Revised fibromyalgia impact questionnaire.

Primary outcome	Baseline–6th week	Baseline–12th week	Baseline–Follow–up	6th–12th week	6th week–Follow–up	12th week–Follow–up
	Estimated mean difference (95% confidence interval)					
Function (0–30)						
Progressive	4.10 (−0.13, 8.33)	5.82 (0.70, 10.94)*	4.29 (−0.40, 8.99)	1.72 (−1.71, 5.15)	0.19 (−2.90, 3.28)	−1.53 (−5.08, 2.02)
Constant	3.84 (−0.39, 8.07)	4.81 (−0.30, 9.93)	6.44 (1.74, 11.14)*	0.97 (−2.46, 4.40)	2.59 (−0.50, 5.69)	1.62 (−1.92, 5.17)
Walking	5.96 (1.73, 10.19)*	6.20 (1.08, 11.32)*	8.30 (3.60, 12.99)*	0.237 (−3.197, 3.671)	2.33 (−0.76, 5.43)	2.09 (−1.45, 5.64)
Impact (0–20)						
Progressive	3.19 (0.02, 6.35)*	3.43 (−0.12, 6.99)	1.96 (−1.38, 5.31)	0.24 (−2.56, 3.05)	−1.25 (−3.70, 1.25)	−1.46 (−4.04, 1.11)
Constant	3.24 (0.07, 6.40)*	4.24 (0.68, 7.80)*	4.90 (1.55, 8.24)*	1 (−1.80, 3.81)	1.65 (−0.81, 4.13)	0.65 (−1.92, 3.23)
Walking	6.21 (3.05, 9.38)*	5.43 (1.87, 8.99)*	7.27 (3.92, 10.62)*	−0.78 (−3.59, 2.02)	1.05 (−1.42, 3.53)	1.83 (−0.74, 4.41)
Symptoms (0–50)						
Progressive	6.43 (1.03, 11.84)*	7.63 (1.56, 13.69)*	4.06 (−2.45, 10.58)	1.19 (−3.39, 5.77)	−2.37 (−7.06, 2.32)	−3.56 (−8.07, 0.95)
Constant	6.78 (1.38, 12.18)*	6.90 (0.84, 12.97)*	7.70 (1.18, 14.22)*	0.12 (−4.45, 4.70)	0.92 (−3.77, 5.61)	0.79 (−3.71, 5.30)
Walking	10.75 (5.356, 16.16)*	10.15 (4.09, 16.21)*	9.25 (2.72, 15.77)*	−0.60 (−5.18, 3.97)	−1.50 (−6.20, 3.18)	−0.90 (−5.41, 3.60)
Total (0–100)						
Progressive	13.73 (2.41, 25.05)*	16.88 (3.17, 30.60)*	10.32 (−3.04, 23.70)	3.15 (−6.50, 12.81)	−3.40 (−12.12, 5.31)	−6.56 (−16.16, 3.04)
Constant	13.86 (2.54, 25.18)*	15.96 (2.25, 29.68)*	19.04 (5.67, 32.42)*	2.09 (−7.55, 11.75)	5.17 (−3.54, 13.90)	3.08 (−6.52, 12.68)
Walking	22.94 (11.62, 34.25)*	21.78 (8.07, 35.50)*	24.82 (11.44, 38.19)*	−1.15 (−10.80, 8.50)	1.88 (−6.84, 10.60)	3.03 (−6.57, 12.63)

*Note:* Baseline: Sample characteristics before treatment. 6th week: Sample characteristics after 12 exercise sessions. 12th week: Sample characteristics after 24 exercise sessions. Follow‐up: Sample characteristics after 3 months without exercise, that is, at the 24th week in relation to the baseline.

*Significant difference observed through linear mixed models (*p* ≤ 0.05).

Although no group reached the traditional minimal clinically important difference of 27 points for the primary outcome, all groups showed reductions of sufficient magnitude to call into question the validity of the minimum clinically important difference. This is based on the observed mean values.

Tables 5 and 6 show the descriptive values and the between‐group comparisons for the secondary outcome at baseline and at week 12 (group vs. group). No variable reached a minimal clinically important difference proposed a priori in the protocol. The groups reported a positive perception of improvement related to treatment, but there was no significant difference between the reports (*p* ≥ 0.05). The majority of each group did not adhere and/or did not respond about adherence after the follow‐up without exercise, but there was also no significant difference between the reports (*p* ≥ 0.05). We observed a significant difference (*p* ≤ 0.05) between the progressive and constant groups in the baseline cutaneous sensory threshold of the supraspinatus muscle (0.75 [95% CI: 0.00; 1.50], *d* = 0.71), indicating that the progressive group had greater sensitivity in this body region. Finally, at week 12, we observed significant differences (*p* ≤ 0.05) between the progressive and walking groups (−6.80 [95% CI: −13.16; −0.44], *d* = 0.75) as well as between the constant and walking groups (−6.97 [95% CI: −13.33; −0.61], *d* = 0.87) in right handgrip strength, suggesting that walking produces smaller strength gains compared to strength training.

**TABLE 5:** Secondary outcome descriptive values.

Secondary outcome	Progressive (*n* = 22)	Constant (*n* = 22)	Walking (*n* = 22)
	Mean (95% confidence interval)		
Widespread pain index (0–19)			
Baseline	9.68 (8.46, 10.90)	11.59 (10.37, 12.81)	11.31 (10.09, 12.53)
12th week	5.81 (4.40, 7.21)	7.22 (5.82, 8.62)	7.26 (5.86, 8.66)
Symptom severity scale (0–12)			
Baseline	6.27 (5.75, 6.79)	6.36 (5.84, 6.88)	6.31 (5.79, 6.83)
12th week	5.06 (4.30, 5.81)	5.47 (4.72, 6.23)	5.29 (4.53, 6.04)
Pittsburgh Sleep quality index (score)			
Subjective sleep quality (0–3)			
Baseline	2.22 (1.94, 2.50)	2.22 (1.94, 2.50)	2.45 (2.17, 2.73)
12th week	1.88 (1.58, 2.18)	1.91 (1.61, 2.20)	1.88 (1.58, 2.18)
Sleep latency (0–3)			
Baseline	2.40 (2.06, 2.75)	2.22 (1.88, 2.56)	2.18 (1.84, 2.52)
12th week	2.32 (2.01, 2.63)	2.22 (1.91, 2.53)	2.09 (1.78, 2.40)
Sleep duration (0–3)			
Baseline	1.86 (1.40, 2.32)	1.50 (1.04, 1.96)	1.40 (0.94, 1.86)
12th week	1.70 (1.34, 2.06)	1.39 (1.03, 1.74)	1.43 (1.07, 1.79)
Habitual sleep efficiency (0–3)			
Baseline	2.40 (1.89, 2.92)	2.59 (2.07, 3.10)	2.09 (1.57, 2.60)
12th week	2.99 (2.94, 3.04)	2.94 (2.89, 2.99)	2.99 (2.94, 3.04)
Sleep disturbances (0–3)			
Baseline	2.45 (2.18, 2.72)	2.50 (2.23, 2.76)	2.45 (2.18, 2.72)
12th week	2.24 (2.02, 2.46)	2.29 (2.07, 2.51)	2.29 (2.06, 2.51)
Use of sleep medication (0–3)			
Baseline	2.13 (1.55, 2.71)	2.00 (1.42, 2.57)	2.04 (1.46, 2.62)
12th week	1.51 (1.00, 2.02)	1.58 (1.08, 2.09)	1.87 (1.37, 2.38)
Daytime dysfunction (0–3)			
Baseline	1.77 (1.43, 2.11)	2.09 (1.75, 2.43)	2.31 (1.97, 2.66)
12th week	1.68 (1.35, 2.01)	1.83 (1.50, 2.17)	1.96 (1.63, 2.29)
Total (0–21)			
Baseline	15.27 (13.68, 16.86)	15.13 (13.54, 16.72)	14.95 (13.36, 16.54)
12th week	14.35 (13.08, 15.62)	14.19 (12.91, 15.46)	14.53 (13.26, 15.81)
Anxiety (HADS, 0–21)			
Baseline	11.90 (10.25, 13.56)	12.59 (10.94, 14.24)	14.36 (12.71, 16.01)
12th week	10.60 (9.04, 12.17)	10.59 (9.03, 12.15)	10.83 (9.27, 12.39)
Depression (HADS, 0–21)			
Baseline	11.04 (9.31, 12.77)	10.86 (9.13, 12.59)	13.09 (11.36, 14.82)
12th week	9.61 (7.94, 11.27)	8.62 (6.96, 10.28)	9.01 (7.35, 10.68)
Wind‐up (TS: Pressure = 2.5 kg)			
0 s (NPRS, 0–10)			
Baseline	4.72 (3.55, 5.89)	4.68 (3.50, 5.85)	5.22 (4.05, 6.40)
12th week	3.76 (2.92, 4.60)	4.49 (3.65, 5.33)	4.52 (3.68, 5.37)
10 s (NPRS, 0–10)			
Baseline	5.79 (4.64, 6.93)	6.13 (4.99, 7.28)	7.13 (5.99, 8.28)
12th week	5.13 (4.21, 6.04)	5.77 (4.86, 6.69)	5.75 (4.83, 6.66)
20 s (NPRS, 0–10)			
Baseline	6.55 (5.44, 7.67)	7.27 (6.15, 8.38)	8.13 (7.02, 9.25)
12th week	5.89 (4.94, 6.83)	6.54 (5.60, 7.48)	6.66 (5.72, 7.60)
30 s (NPRS, 0–10)			
Baseline	7.04 (6.02, 8.06)	8.00 (6.98, 9.01)	8.40 (7.39, 9.42)
12th week	6.64 (5.64, 7.63)	7.02 (6.02, 8.02)	6.94 (5.94, 7.93)
Conditioned pain modulation (≥ 100)			
Baseline	154.12 (105.72, 202.52)	115.89 (67.50, 164.29)	122.45 (74.05, 170.85)
12th week	185.09 (106.81, 263.37)	118.95 (40.67, 197.23)	106.11 (27.83, 184.39)
Cutaneous sensory threshold (gf)			
Trapezius			
Baseline	1.21 (0.57, 1.86)	1.43 (0.78, 2.07)	0.93 (0.28, 1.57)
12th week	1.32 (0.72, 1.91)	1.25 (0.66, 1.85)	1.45 (0.86, 2.06)
Supraspinatus			
Baseline	0.76 (0.33, 1.19)	1.51 (1.08, 1.94)	1.16 (0.73, 1.59)
12th week	1.20 (0.86, 1.53)	0.94 (0.60, 1.28)	1.07 (0.73, 1.41)
Sternocleidomastoid			
Baseline	0.22 (−0.15, 0.60)	0.83 (0.45, 1.21)	0.58 (0.20, 0.96)
12th week	0.42 (0.16, 0.68)	0.53 (0.27, 0.78)	0.74 (0.48, 1.00)
Walking ability (6MWT, m)			
Baseline	455.71 (416.23, 495.19)	408.30 (368.82, 447.78)	395.33 (355.84, 434.81)
12th week	455.10 (436.43, 473.76)	455.40 (436.73, 474.07)	446.02 (427.35, 464.68)
Knee extension (dynamometry)			
Peak torque (Nm)			
Baseline	80.42 (68.29, 92.54)	93.33 (81.49, 105.18)	81.70 (69.85, 93.54)
12th week	102.71 (96.97, 108.46)	96.14 (90.53, 101.76)	96.63 (91.02, 102.25)
Total work (J)			
Baseline	691.25 (584.89, 797.60)	785.36 (681.45, 889.27)	688.16 (584.26, 792.07)
12th week	875.89 (827.55, 924.24)	814.17 (766.94, 861.40)	817.94 (770.71, 865.17)
Strength (W)			
Baseline	53.70 (44.93, 62.46)	63.64 (55.08, 72.21)	54.94 (46.38, 63.50)
12th week	69.17 (64.59, 73.74)	63.84 (59.38, 68.31)	65.01 (60.54, 69.48)
Knee flexion (dynamometry)			
Peak torque (Nm)			
Baseline	23.45 (16.94, 29.954)	29.98 (23.63, 36.338)	25.33 (18.97, 31.684)
12th week	32.94 (28.84, 37.045)	28.54 (24.54, 32.556)	31.84 (27.83, 35.853)
Total work (J)			
Baseline	190.14 (133.50, 246.789)	238.87 (183.53, 294.215)	196.69 (141.35, 252.035)
12th week	283.82 (243.65, 323.991)	240.30 (201.05, 279.551)	271.72 (232.48, 310.972)
Strength (W)			
Baseline	14.62 (10.05, 19.19)	18.76 (14.30, 23.235)	15.65 (11.19, 20.124)
12th week	21.28 (18.17, 24.39)	17.95 (14.91, 20.99)	20.40 (17.36, 23.446)
Right handgrip strength (kg)			
Baseline	20 (14.59, 25.41)	18.76 (13.35, 24.17)	21.49 (16.08, 26.90)
12th week	23.96 (20.30, 27.61)	24.13 (20.47, 27.78)	17.15 (13.50, 20.81)
Left handgrip strength (kg)			
Baseline	21.25 (15.90, 26.61)	20.07 (14.72, 25.42)	21.87 (16.52, 27.23)
12th week	26.84 (22.70, 30.97)	26.62 (22.49, 30.75)	20.76 (16.62, 24.89)
Global perceived effect (−5, +5)			
6th week	1.71 (0.97, 2.45)	1.59 (0.85, 2.32)	1.69 (0.95, 2.43)
12th week	1.86 (0.85, 2.87)	0.72 (−0.28, 1.73)	1.04 (0.04, 2.05)
Follow‐up (24th week)	1.31 (0.50, 2.11)	1.07 (0.27, 1.88)	0.57 (−0.22, 1.38)
Exercise adherence (24th week)			
Not (*n* = 25/66)	10 (45.5%)	7 (31.8%)	8 (36.4%)
Yes (*n* = 14/66)	5 (22.7%)	5 (22.7%)	4 (18.2%)
Unanswered (*n* = 27/66)	7 (31.8%)	10 (45.5%)	10 (45.5%)

*Note:* HADS: Hospital Anxiety and Depression Scale. NPRS: Numerical Pain Rating Scale. 6MWT: Six‐Minute Walk Test. TS: Temporal summation at a constant pressure of 2.5 kg for 30 s. Baseline: Sample characteristics before treatment. 6th week: Sample characteristics after 12 exercise sessions. 12th week: Sample characteristics after 24 exercise sessions. Follow‐up: Sample characteristics after 3 months without exercise, that is, at the 24th week in relation to the baseline.

**TABLE 6:** Secondary outcome between‐group comparisons (group vs. group).

Secondary outcome	Progressive–Constant	Progressive–Walking	Constant–Walking
	Estimated mean difference (95% confidence interval)		
Widespread pain index (0–19)			
Baseline	1.90 (−0.21, 4.03)	1.63 (−0.48, 3.76)	−0.27 (−2.39, 1.85)
12th week	1.41 (−1.02, 3.85)	1.45 (−0.98, 3.89)	0.04 (−2.39, 2.48)
Symptom severity scale (0–12)			
Baseline	0.09 (−0.81, 0.99)	0.04 (−0.86, 0.95)	−0.04 (−0.95, 0.86)
12th week	0.41 (−0.89, 1.72)	0.22 (−1.08, 1.54)	−0.18 (−1.50, 1.12)
Pittsburgh Sleep quality index (score)			
Subjective sleep quality (0–3)			
Baseline	0.66 (−0.48, 0.48)	0.22 (−0.26, 0.71)	0.22 (−0.26, 0.71)
12th week	0.02 (−0.49, 0.54)	−0.88 (−0.51, 0.51)	−0.02 (−0.54, 0.49)
Sleep latency (0–3)			
Baseline	−0.18 (−0.77, 0.41)	−0.22 (−0.82, 0.36)	−0.04 (−0.64, 0.54)
12th week	−0.09 (−0.63, 0.44)	−0.22 (−0.76, 0.31)	−0.13 (−0.66, 0.41)
Sleep duration (0–3)			
Baseline	−0.36 (−1.16, 0.43)	−0.45 (−1.25, 0.34)	−0.09 (−0.89, 0.71)
12th week	−0.31 (−0.93, 0.30)	−0.27 (−0.89, 0.35)	0.04 (−0.57, 0.66)
Habitual sleep efficiency (0–3)			
Baseline	0.18 (−0.71, 1.08)	−0.31 (−1.21, 0.58)	−0.50 (−1.39, 0.39)
12th week	−0.04 (−0.14, 0.04)	0.98 (−0.09, 0.09)	0.04 (−0.04, 0.14)
Sleep disturbances (0–3)			
Baseline	0.04 (−0.41, 0.50)	0.031 (−0.46, 0.46)	−0.04 (−0.50, 0.41)
12th week	0.05 (−0.33, 0.44)	0.04 (−0.34, 0.43)	−0.00 (−0.39, 0.38)
Use of sleep medication (0–3)			
Baseline	−0.13 (−1.14, 0.86)	−0.09 (−1.09, 0.91)	0.04 (−0.95, 1.05)
12th week	0.07 (−0.80, 0.95)	0.36 (−0.51, 1.24)	0.28 (−0.59, 1.17)
Daytime dysfunction (0–3)			
Baseline	0.31 (−0.27, 0.91)	0.54 (−0.04, 1.14)	0.22 (−0.36, 0.82)
12th week	0.15 (−0.42, 0.72)	0.27 (−0.30, 0.84)	0.12 (−0.45, 0.69)
Total (0–21)			
Baseline	−0.13 (−2.90, 2.62)	−0.31 (−3.08, 2.44)	−0.18 (−2.94, 2.58)
12th week	−0.16 (−2.38, 2.05)	0.18 (−2.03, 2.40)	0.34 (−1.87, 2.56)
Anxiety (HADS, 0–21)			
Baseline	0.68 (−2.19, 3.55)	2.45 (−0.41, 5.32)	1.77 (−1.10, 4.64)
12th week	−0.01 (−2.72, 2.70)	0.22 (−2.48, 2.94)	0.24 (−2.47, 2.95)
Depression (HADS, 0–21)			
Baseline	−0.18 (−3.19, 2.83)	2.04 (−0.96, 5.05)	2.22 (−0.78, 5.23)
12th week	−0.98 (−3.87, 1.90)	−0.59 (−3.48, 2.30)	0.39 (−2.50, 3.28)
Wind–up (TS: Pressure = 2.5 kg)			
0 s (NPRS, 0–10)			
Baseline	−0.04 (−2.08, 2.00)	0.50 (−1.54, 2.54)	0.54 (−1.50, 2.59)
12th week	0.73 (−0.73, 2.19)	0.76 (−0.70, 2.23)	0.03 (−1.43, 1.50)
10 s (NPRS, 0–10)			
Baseline	0.34 (−1.64, 2.33)	1.34 (−0.64, 3.33)	1.00 (−0.99, 2.99)
12th week	0.64 (−0.94, 2.23)	0.62 (−0.96, 2.21)	−0.02 (−1.61, 1.56)
20 s (NPRS, 0–10)			
Baseline	0.71 (−1.22, 2.65)	1.57 (−0.36, 3.51)	0.86 (−1.07, 2.80)
12th week	0.65 (−0.98, 2.29)	0.77 (−0.86, 2.41)	0.12 (−1.51, 1.75)
30 s (NPRS, 0–10)			
Baseline	0.95 (−0.82, 2.72)	1.36 (−0.41, 3.13)	0.40 (−1.36, 2.18)
12th week	0.38 (−1.35, 2.12)	0.30 (−1.43, 2.04)	−0.08 (−1.82, 1.65)
Conditioned pain modulation (≥ 100)			
Baseline	−38.22 (−122.47, 46.01)	−31.67 (−115.91, 52.57)	6.55 (−77.69, 90.79)
12th week	−66.13 (−202.39, 70.12)	−78.97 (−215.23, 57.28)	−12.84 (−149.10, 123.41)
Cutaneous sensory threshold (gf)			
Trapezius			
Baseline	0.21 (−0.91, 1.33)	−0.28 (−1.41, 0.83)	−0.50 (−1.62, 0.62)
12th week	−0.06 (−1.09, 0.97)	0.14 (−0.89, 1.18)	0.20 (−0.83, 1.24)
Supraspinatus			
Baseline	0.75 (0.00, 1.50)*	0.40 (−0.35, 1.15)	−0.35 (−1.10, 0.40)
12th week	−0.25 (−0.84, 0.33)	−0.12 (−0.71, 0.46)	0.13 (−0.46, 0.72)
Sternocleidomastoid			
Baseline	0.60 (−0.05, 1.26)	0.35 (−0.30, 1.01)	−0.24 (−0.90, 0.41)
12th week	0.10 (−0.34, 0.55)	0.31 (−0.13, 0.76)	0.21 (−0.23, 0.66)
Walking ability (6MWT, m)			
Baseline	−47.40 (−116.13, 21.31)	−60.38 (−129.10, 8.34)	−12.97 (−81.69, 55.74)
12th week	0.30 (−32.18, 32.79)	−9.08 (−41.57, 23.41)	−9.38 (−41.87, 23.10)
Knee extension (dynamometry)			
Peak torque (Nm)			
Baseline	12.91 (−7.89, 33.72)	1.28 (−19.52, 22.08)	−11.63 (−32.20, 8.92)
12th week	−6.57 (−16.43, 3.28)	−6.08 (−15.94, 3.78)	0.49 (−9.25, 10.23)
Total work (J)			
Baseline	94.11 (−88.41, 276.64)	−3.08 (−185.61, 179.44)	−97.19 (−277.59, 83.19)
12th week	−61.72 (−144.69, 21.24)	−57.95 (−140.92, 25.01)	3.77 (−78.22, 85.77)
Strength (W)			
Baseline	9.94 (−5.09, 24.98)	1.24 (−13.79, 16.28)	−8.70 (−23.56, 6.16)
12th week	−5.32 (−13.16, 2.52)	−4.15 (−12.00, 3.69)	1.16 (−6.59, 8.92)
Knee flexion (dynamometry)			
Peak torque (Nm)			
Baseline	6.53 (−4.62, 17.69)	1.87 (−9.28, 13.04)	−4.65 (−15.68, 6.37)
12th week	−4.39 (−11.43, 2.64)	−1.09 (−8.13, 5.94)	3.29 (−3.65, 10.25)
Total work (J)			
Baseline	48.72 (−48.48, 145.93)	6.54 (−90.66, 103.75)	−42.18 (−138.25, 53.89)
12th week	−43.51 (−112.45, 25.42)	−12.09 (−81.03, 56.84)	31.42 (−36.71, 99.55)
Strength (W)			
Baseline	4.14 (−3.69, 11.99)	1.03 (−6.80, 8.88)	−3.11 (−10.86, 4.64)
12th week	−3.32 (−8.67, 2.01)	−0.87 (−6.21, 4.46)	2.45 (−2.82, 7.73)
Right handgrip strength (kg)			
Baseline	−1.24 (−10.65, 8.17)	1.49 (−7.92, 10.90)	2.73 (−6.68, 12.15)
12th week	0.16 (−6.19, 6.52)	−6.80 (−13.16, −0.44)*	−6.97 (−13.33, −0.61)*
Left handgrip strength (kg)			
Baseline	−1.18 (−10.50, 8.13)	0.62 (−8.69, 9.93)	1.80 (−7.51, 11.12)
12th week	−0.21 (−7.40, 6.97)	−6.08 (−13.27, 1.11)	−5.86 (−13.05, 1.32)
Global perceived effect (−5, +5)			
6th week	−0.12 (−1.40, 1.16)	−0.01 (−1.30, 1.27)	0.10 (−1.17, 1.39)
12th week	−1.14 (−2.89, 0.61)	−0.81 (−2.57, 0.93)	0.32 (−1.43, 2.07)
Follow–up (24th week)	−0.23 (−1.63, 1.17)	−0.73 (−2.13, 0.67)	−0.50 (−1.90, 0.90)
Exercise adherence (24th week)			
No	13.7%	9.1%	4.6%
Yes	0	4.5%	4.5%
Unanswered	13.7%	13.7%	0

*Note:* HADS: Hospital Anxiety and Depression Scale. NPRS: Numerical Pain Rating Scale. 6MWT: Six‐Minute Walk Test. TS: Temporal summation at a constant pressure of 2.5 kg for 30 s. Baseline: Sample characteristics before treatment. 6th week: Sample characteristics after 12 exercise sessions. 12th week: Sample characteristics after 24 exercise sessions. Follow‐up: Sample characteristics after 3 months without exercise, that is, at the 24th week in relation to the baseline.

*Significant difference observed through linear mixed models (*p* ≤ 0.05).

Table 7 describes the between‐group delta comparisons for the secondary outcome baseline–week 12 (time–time vs. group–group). We observed a significant difference (*p* ≤ 0.05) between the progressive and constant groups in supraspinatus muscle cutaneous sensory threshold (−1.00 [95% CI: −1.81; −0.19], *d* = 0.95) and knee extension strength (−14.61 [95% CI: −29.10; −0.12], *d* = 0.84), confirming that the progressive group had greater sensitivity and achieved greater strength gains. The deltas were also confirmed by significant difference (*p* ≤ 0.05) that walking produced smaller gains in right handgrip strength compared to progressive (−8.29 [95% CI: −16.37; −0.22], *d* = 0.79) or constant (−9.70 [95% CI: −17.78; −1.63], *d* = 0.89) intensity strength training.

**TABLE 7:** Between‐group delta comparisons: time–time versus group–group (secondary outcome).

Secondary outcome (Δ)	Progressive–Constant	Progressive–Walking	Constant–Walking
	Estimated mean difference (95% confidence interval)		
Baseline–12th week			
Widespread pain index (0–19)	−0.49 (−3.00, 2.00)	−0.18 (−2.68, 2.32)	0.31 (−2.19, 2.82)
Symptom severity scale (0–12)	0.32 (−1.11, 1.75)	0.18 (−1.25, 1.61)	−0.14 (−1.57, 1.29)
Pittsburgh Sleep quality index (score)			
Subjective sleep quality (0–3)	0.02 (−0.61, 0.67)	−0.22 (−0.87, 0.41)	−0.25 (−0.89, 0.39)
Sleep latency (0–3)	0.08 (−0.48, 0.65)	0.05 (−0.57, 0.57)	−0.08 (−0.65, 0.48)
Sleep duration (0–3)	0.04 (−0.66, 0.76)	0.18 (−0.53, 0.89)	0.13 (−0.58, 0.85)
Habitual sleep efficiency (0–3)	−0.23 (−1.13, 0.67)	0.31 (−0.58, 1.22)	0.54 (−0.35, 1.45)
Sleep disturbances (0–3)	0.00 (−0.51, 0.52)	0.04 (−0.47, 0.56)	0.04 (−0.48, 0.56)
Use of sleep medication (0–3)	0.21 (−0.86, 1.28)	0.45 (−0.62, 1.53)	0.24 (−0.83, 1.32)
Daytime dysfunction (0–3)	−0.16 (−0.85, 0.52)	−0.27 (−0.96, 0.41)	−0.10 (−0.79, 0.58)
Total (0–21)	−0.02 (−3.11, 3.06)	0.50 (−2.59, 3.59)	0.52 (−2.56, 3.61)
Anxiety (HADS, 0–21)	−0.69 (−3.57, 2.18)	−2.22 (−5.11, 0.65)	−1.53 (−4.41, 1.35)
Depression (HADS, 0–21)	−0.80 (−3.95, 2.34)	−2.63 (−5.78, 0.51)	−1.83 (−4.98, 1.31)
Wind–up (TS: Pressure = 2.5 kg)			
0 s (NPRS, 0–10)	0.77 (−0.96, 2.51)	0.26 (−1.48, 2.00)	−0.51 (−2.25, 1.22)
10 s (NPRS, 0–10)	0.30 (−1.41, 2.01)	−0.72 (−2.43, 0.98)	−1.02 (−2.73, 0.68)
20 s (NPRS, 0–10)	−0.05 (−1.80, 1.69)	−0.80 (−2.55, 0.95)	−0.74 (−2.49, 1.00)
30 s (NPRS, 0–10)	−0.56 (−2.31, 1.18)	−1.06 (−2.80, 0.68)	−0.49 (−2.24, 1.25)
Conditioned pain modulation (≥ 100)	−27.90 (−91.04, 35.23)	−47.30 (−110.44, 15.83)	−19.39 (−82.53, 43.74)
Cutaneous sensory threshold (gf)			
Trapezius	−0.27 (−1.77, 1.22)	0.43 (−1.06, 1.93)	0.70 (−0.79, 2.20)
Supraspinatus	−1.00 (−1.81, −0.19)*	−0.52 (−1.33, 0.28)	0.48 (−0.32, 1.28)
Sternocleidomastoid	−0.50 (−1.29, 0.29)	−0.03 (−0.83, 0.76)	0.46 (−0.33, 1.26)
Walking ability (6MWT, m)	47.71 (−13.96, 109.38)	51.30 (−10.37, 112.97)	3.58 (−58.08, 65.26)
Knee extension (dynamometry)			
Peak torque (Nm)	−18.46 (−37.75, 0.83)	−6.33 (−25.62, 12.95)	12.12 (−7.16, 31.41)
Total work (J)	−146.80 (−317.70, 24.08)	−45.83 (−216.73, 125.05)	100.97 (−69.92, 271.86)
Strength (W)	−14.61 (−29.10, −0.12)*	−4.74 (−19.23, 9.73)	9.86 (−4.61, 24.35)
Knee flexion (dynamometry)			
Peak torque (Nm)	−9.77 (−19.98, 0.43)	−1.82 (−12.03, 8.39)	7.95 (−2.26, 18.16)
Total work (J)	−82.31 (−175.80, 11.17)	−8.71 (−102.20, 84.77)	73.60 (−19.88, 167.09)
Strength (W)	−6.79 (−14.29, 0.70)	−1.23 (−8.73, 6.26)	5.56 (−1.93, 13.06)
Right handgrip strength (kg)	1.40 (−6.66, 9.48)	−8.29 (−16.37, −0.22)*	−9.70 (−17.78, −1.63)*
Left handgrip strength (kg)	0.96 (−7.12, 9.06)	−6.70 (−14.79, 1.39)	−7.66 (−15.76, 0.42)
Global perceived effect (−5, +5)			
6th–12th week	−1.02 (−2.87, 0.83)	−0.80 (−2.65, 1.05)	0.21 (−1.63, 2.07)
6th week–Follow–up	−0.11 (−1.45, 1.23)	−0.71 (−2.06, 0.63)	−0.60 (−1.95, 0.74)
12th week–Follow–up	0.91 (−0.89, 2.72)	0.08 (−1.72, 1.89)	−0.82 (−2.63, 0.98)

*Note:* HADS: Hospital Anxiety and Depression Scale. NPRS: Numerical Pain Rating Scale. 6MWT: Six‐Minute Walk Test. TS: Temporal summation at a constant pressure of 2.5 kg for 30 s. Baseline: Sample characteristics before treatment. 6th week: Sample characteristics after 12 exercise sessions. 12th week: Sample characteristics after 24 exercise sessions. Follow‐up: Sample characteristics after 3 months without exercise, that is, at the 24th week in relation to the baseline.

*Significant difference observed through linear mixed models (*p* ≤ 0.05).

Table 8 shows the within‐group comparisons for the secondary outcomes. Significant differences were observed in all groups (*p* ≤ 0.05, *d* > 0.5) in terms of sleep quality, anxiety, depression, wind‐up mechanism, conditioned pain modulation, cutaneous sensory threshold, musculoskeletal performance, walking ability, and perceived improvement. However, none of the variables achieved a minimal clinically important difference.

**TABLE 8:** Secondary outcome within‐group comparisons (time vs. time).

Secondary outcome (Δ)	Progressive	Constant	Walking
	Estimated mean difference (95% confidence interval)		
Baseline–12th week			
Widespread pain index (0–19)	3.87 (2.43, 5.31)*	4.36 (2.92, 5.80)*	4.05 (2.61, 5.49)*
Symptom severity scale (0–12)	1.21 (0.38, 2.03)*	0.88 (0.06, 1.71)*	1.02 (0.20, 1.85)*
Pittsburgh Sleep quality index (score)			
Subjective sleep quality (0–3)	0.34 (−0.02, 0.71)	0.31 (−0.05, 0.68)	0.57 (0.20, 0.93)*
Sleep latency (0–3)	0.08 (−0.24, 0.41)	0.00 (−0.32, 0.33)	0.08 (−0.24, 0.41)
Sleep duration (0–3)	0.15 (−0.25, 0.56)	0.10 (−0.30, 0.52)	−0.02 (−0.43, 0.38)
Habitual sleep efficiency (0–3)	−0.58 (−1.10, −0.06)*	−0.35 (−0.87, 0.16)	−0.90 (−1.42, −0.38)*
Sleep disturbances (0–3)	0.21 (−0.09, 0.51)	0.20 (−0.09, 0.50)	0.16 (−0.13, 0.46)
Use of sleep medication (0–3)	0.62 (0.00, 1.24)*	0.41 (−0.20, 1.03)	0.16 (−0.45, 0.78)
Daytime dysfunction (0–3)	0.08 (−0.31, 0.47)	0.25 (−0.14, 0.64)	0.35 (−0.03, 0.75)
Total (0–21)	0.91 (−0.85, 2.69)	0.94 (−0.83, 2.71)	0.41 (−1.35, 2.19)
Anxiety (HADS, 0–21)	1.30 (−0.35, 2.95)	1.99 (0.33, 3.65)*	3.52 (1.87, 5.18)*
Depression (HADS, 0–21)	1.43 (−0.37, 3.24)	2.23 (0.42, 4.04)*	4.07 (2.26, 5.88)*
Wind–up (TS: Pressure = 2.5 kg)			
0 s (NPRS, 0–10)	0.95 (−0.04, 1.95)	0.18 (−0.81, 1.18)	0.69 (−0.30, 1.69)
10 s (NPRS, 0–10)	0.66 (−0.32, 1.64)	0.36 (−0.62, 1.34)	1.38 (0.40, 2.36)*
20 s (NPRS, 0–10)	0.66 (−0.33, 1.67)	0.72 (−0.28, 1.73)	1.46 (0.46, 2.47)*
30 s (NPRS, 0–10)	0.40 (−0.59, 1.41)	0.97 (−0.02, 1.97)	1.46 (0.46, 2.47)*
Conditioned pain modulation (≥ 100)	−30.96 (−67.23, 5.31)	−3.05 (−39.32, 33.21)	16.34 (−19.93, 52.61)
Cutaneous sensory threshold (gf)			
Trapezius	−0.10 (−0.96, 0.75)	0.17 (−0.69, 1.03)	−0.53 (−1.39, 0.32)
Supraspinatus	−0.43 (−0.90, 0.02)	0.56 (0.10, 1.03)*	0.08 (−0.37, 0.55)
Sternocleidomastoid	−0.20 (−0.65, 0.25)	0.30 (−0.15, 0.76)	−0.16 (−0.62, 0.29)
Walking ability (6MWT, m)	0.61 (−34.82, 36.04)	−47.10 (−82.53, −11.67)*	−50.69 (−86.12, −15.25)*
Knee extension (dynamometry)			
Peak torque (Nm)	−22.29 (−33.66, −10.92)*	−2.80 (−13.92, 8.30)	−14.93 (−26.04, −3.82)*
Total work (J)	−184.64 (−285.40, −83.88)*	−28.80 (−127.24, 69.63)	−129.77 (−228.21, −31.33)*
Strength (W)	−15.47 (−24.02, −6.91)*	−0.20 (−8.55, 8.15)	−10.06 (−18.42, −1.71)*
Knee flexion (dynamometry)			
Peak torque (Nm)	−9.49 (−15.39, −3.59)*	1.43 (−4.32, 7.19)	−6.51 (−12.27, −0.75)*
Total work (J)	−93.67 (−147.83, −39.51)*	−1.42 (−54.34, 51.48)	−75.02 (−127.94, −22.11)*
Strength (W)	−6.65 (−11.03, −2.28)*	0.81 (−3.45, 5.08)	−4.74 (−9.01, −0.47)*
Right handgrip strength (kg)	−3.96 (−8.59, 0.67)	−5.37 (−10.00, −0.73)*	4.33 (−0.30, 8.97)
Left handgrip strength (kg)	−5.58 (−10.23, −0.93)*	−6.55 (−11.20, −1.90)*	1.11 (−3.53, 5.76)
Global perceived effect (−5, +5)			
6th–12th week	−0.15 (−1.46, 1.15)	0.86 (−0.44, 2.17)	0.64 (−0.66, 1.95)
6th week–Follow–up	0.40 (−0.55, 1.35)	0.51 (−0.44, 1.46)	1.11 (0.16, 2.07)*
12th week–Follow–up	0.55 (−0.72, 1.83)	−0.35 (−1.63, 0.92)	0.47 (−0.80, 1.74)

*Note:* HADS: Hospital Anxiety and Depression Scale. NPRS: Numerical Pain Rating Scale. 6MWT: Six‐Minute Walk Test. TS: Temporal summation at a constant pressure of 2.5 kg for 30 s. Baseline: Sample characteristics before treatment. 6th week: Sample characteristics after 12 exercise sessions. 12th week: Sample characteristics after 24 exercise sessions. Follow‐up: Sample characteristics after 3 months without exercise, that is, at the 24th week in relation to the baseline.

*Significant difference observed through linear mixed models (*p* ≤ 0.05).

## 4 Discussion

### 4.1 Primary Outcome Discussion: Baseline, 6th, 12th Week, and Follow‐Up

Although the total score of the revised fibromyalgia impact questionnaire (0–100) is the most commonly used score in fibromyalgia studies, there is still controversy about the real minimal clinically important difference in identifying the effectiveness of an intervention. Bennett et al. suggested a 14% reduction in the total score of the traditional questionnaire (Bennett et al. 2009), while Surendran and Mithun suggested a 45.5% reduction in the revised questionnaire (Surendran and Mithun 2018). Recently, Lee highlighted some limitations in the measurement properties related to the sum of the domains of the revised questionnaire and emphasised that there is still no consistent data on its minimal clinically important difference (Lee 2021). In light of this finding and considering the 27‐item proposal (Surendran and Mithun 2018), our results did not reach the minimum clinically important difference at any of the time points evaluated (baseline, 6, 12, and/or 24 weeks), indicating that there is no better exercise than the other.

When analysing the comparisons between the impact domains of the instrument, walking appears to be superior to progressive intensity strength training at baseline–follow‐up and at week 12–follow‐up. However, there is still no scientific evidence that this difference is clinically important. In addition, these groups were different at baseline and the a priori predicted primary outcome did not account for analyses by domain. This finding supports the literature recommending this type of exercise for people with fibromyalgia, as Casanova‐Rodríguez et al. (2025) found that most of the studies summarised in a systematic review showed significant differences in favour of aerobic exercise (−0.49 [95% CI: −0.90, −0.08]), with moderate to low heterogeneity in the analyses of subgroups of people with fibromyalgia.

Recently, substantial advances have been made in the exercise literature for fibromyalgia, with several high‐quality trials and reviews having been published in recent years. Rodriguez‐Almagro et al. (2023) demonstrated in their meta‐analysis that both aerobic and strengthening protocols consistently reduce pain and improve physical function, with effect sizes comparable across modalities. Similarly, a recent network meta‐analysis by Rodríguez‐Domínguez et al. showed that a wide range of therapeutic exercise modalities—including resistance training, aquatic exercise, and mind–body approaches—are effective in reducing pain intensity, with no single modality consistently outperforming the others (Rodríguez‐Domínguez et al. 2025).

In this context, Zure et al. (2025) reported that low‐load blood‐flow‐restriction strength training produces clinically meaningful improvements while requiring reduced mechanical stress—an important consideration for patients with low exercise tolerance. These contemporary findings also highlight the growing use of digital adherence strategies, which have shown promising effects on symptom burden and continuity of exercise practice. Taken together, this evolving body of evidence helps explain our results: the absence of between‐group differences, the non‐achievement of the 27 points minimal clinically important difference, and the unexpected superiority of walking in certain pain domains are all consistent with recent data suggesting that symptom improvement may be driven more by non‐specific exercise effects, patient tolerance, and behavioural engagement than by the specific training modality itself.

### 4.2 Secondary Outcome Discussion: Baseline, 12h week, and Follow‐Up

The comparisons of secondary outcomes confirm that no exercise is superior to the other, as no analysis reached a minimal clinically important difference between‐groups. However, it is important to note the significant difference in right handgrip strength between the progressive and walking groups, as well as between the constant and walking groups. Although it is plausible to conclude that walking produces smaller strength gains compared to strength training, it must be considered that higher scores on the total score of the revised fibromyalgia impact questionnaire are associated with lower handgrip strength performance in people with fibromyalgia (Salaffi et al. 2020). Our results do not show significant differences among the three total scores, but it should be considered that the walking group showed higher descriptive values of this variable during the intervention.

It should also be noted that the progressive intensity strength training group showed greater musculoskeletal performance compared with the constant intensity group in terms of knee extension strength as assessed by isokinetic dynamometry. In an evaluation similar to our study, Tavares et al. (2020) showed that women with fibromyalgia had 7.04% lower knee extension strength than healthy women. Our results suggest that 24 sessions of progressive intensity strength training increase the musculoskeletal performance of people with fibromyalgia so that knee extension strength becomes equal to or greater than that of physically inactive healthy women, as we observed an increase of 22.36% after the intervention. This finding also confirms the overload principle in people with fibromyalgia, whose biological plausibility indicates that the muscle adapts to withstand greater resistance (Lievens et al. 2021).

Our study had a dropout rate of 28.78% distributed among the three groups, as demonstrated in the results. Although this loss of sample prevents us from evaluating the eighth item of the PEDro scale (Pontes‐Silva, Dibai‐Filho, et al. 2023a), these results are consistent with other clinical trials that have studied people with fibromyalgia undergoing non‐pharmacological treatment. Such as, Sousa et al. reported sample loss of 24.44% (Sousa et al. 2024), Avila et al. (2017) 28.57%, and Trevisan et al. (2015) 68%. In fact, the pattern of dropout from interventions in people with fibromyalgia is still poorly understood scientifically, although more than three thousand people with fibromyalgia were analysed in a systematic review of dropout from exercise interventions (Vancampfort et al. 2024).

In any case, this dropout rate allows us to observe the behaviour of people with fibromyalgia under a clinical treatment routine. Therefore, intention‐to‐treat analysis is essential in clinical trials such as ours, and all patients in all groups must be maintained until the end of treatment, regardless of what happens to each of them (Nagel et al. 2020). Studies that violate the intention‐to‐treat principle produce results without external validity, such as the study by Alves et al. (2024) that excluded five participants, or the study by Serrano et al. that assigned 22 people to a group and analysed 24 after the intervention (Serrano et al. 2022).

Although the between‐group comparison of dropout rates was not statistically significant, the overall attrition rate in this trial was 28.8% (19 out of 66 participants), and the attrition rate was particularly high in the constant‐intensity strength group (40.9%). These results represent important methodological limitations. High attrition reduces statistical power, increases uncertainty around effect estimates, and may introduce attrition bias, even when applying intention‐to‐treat principles. Furthermore, differential dropout, especially concentrated in a single intervention arm, may reflect lower tolerability, acceptability, or perceived benefit of that modality and deserves careful consideration. These patterns are consistent with the well‐documented difficulty of retaining individuals with fibromyalgia in long‐term exercise programs. Nonetheless, these patterns limit the robustness and generalisability of our findings.

Another variable that has been widely discussed among fibromyalgia researchers is exercise adherence (Han et al. 2024). According to Serrat et al., exercise adherence in people with fibromyalgia can be explained by adjusting the intensity: if the intensity is too low, the exercise becomes ineffective and the patient becomes demotivated; in contrast, if the intensity is too high, the overload also demotivates the patient and reduces adherence. Thus, the ideal would be a balance between minimum and maximum, combined with contact with patients (by phone or e‐mail) during the intervention period (Serrat et al. 2021). Our results refute this assumption because we maintained individual contact with our patients throughout the treatment and tested different exercise intensities, that is, progressive or constant intensity, strength training or walking. The results did not differ from those described in the literature.

The literature also highlights the difficulties of long‐term treatment of fibromyalgia (Häuser et al. 2015). Hauser et al. described that the positive results achieved during non‐pharmacological treatment are often lost in the first 12 months at the end of the intervention. In addition, the poor adherence to people with fibromyalgia is a challenge for clinicians and researchers (Häuser et al. 2015). Our results confirm this information and add new evidence. Although all groups in our study reported a positive perception of improvement related to the treatment, the majority of each group did not adhere to the treatment and/or did not respond about adherence after the 3‐month follow‐up without exercise.

Furthermore, we observed that the positive results obtained during treatment seem to stabilise after the sixth week, which may be one of the reasons why patients begin to dropout. To the best of our knowledge, there is still no explanation for this phenomenon, but there is evidence that people with fibromyalgia who receive social support have greater fibromyalgia knowledge and cope better with the adversities of symptoms. Therefore, there is a need for studies that examine the relationship between social support and exercise adherence in people with fibromyalgia.

### 4.3 Study Strengths: Clinical and Scientific Implications

The results of this study have relevant clinical applicability in the treatment of people with fibromyalgia. Although the differences observed did not reach an adequate level of clinical relevance, it is important to emphasise that most results do not yet represent a minimal clinically important difference that has been consolidated in the literature. Nevertheless, knowing that walking or strength training improves the quality of life of these people, the health care professional can choose the type of exercise that meets the patient's preference, according to evidence‐based health recommendations (Aldington and Eccleston 2019).

Our results also reinforce the applicability of the second law of the thermodynamics to musculoskeletal rehabilitation, where the efficiency of muscle work resulting from exercise is the determining factor in the generation of entropy in the homoeostasis of people with fibromyalgia (Koch and Britton 2022). Since the rate of entropy generation increases with the amount of physical exercise, the biological adaptations resulting from the random disorder balance the outcomes related to fibromyalgia symptoms (Koch and Britton 2022). This explains why people with fibromyalgia who underwent 24 sessions of physical exercise showed improvements in sleep quality, anxiety, depression, wind‐up mechanism, conditioned pain modulation, cutaneous sensory threshold, musculoskeletal performance, walking ability, and perception of improvement.

Importantly, the commonly cited minimum clinically important difference of 27 points for the Revised Fibromyalgia Impact Questionnaire is based on absolute rather than relative change, creating substantial interpretive limitations. Since this threshold does not account for baseline severity, patients with low or moderate initial Revised Fibromyalgia Impact Questionnaire scores cannot achieve a clinically significant improvement, even when they experience a meaningful reduction in symptoms. Furthermore, the requirement of a reduction of approximately one‐third in total symptom burden is unusually high compared to the minimum clinically important difference criteria used for other clinical measures. Consequently, applying this fixed cutoff may underestimate true clinical improvement and minimise the substantial effect sizes observed in our sample. Therefore, interpretations based solely on these 27‐point thresholds should be approached with caution. Complementary approaches, such as percentage change or standardised effect sizes, may provide a more accurate representation of meaningful clinical benefit.

Finally, these findings support the hypothesis that the different types of exercise produce similar biological adaptations. Some researchers in the exercise science field argue that health outcomes resulting from exercise, such as musculoskeletal rehabilitation, are not related to the type of physical exercise performed, but rather to the amount of effort applied (i.e., total work). This means that in order to compare different types of exercise, it is crucial that the parameters of the proposed exercises are the same between‐groups, namely intensity, total physical effort, and actual duration of the exercise session. However, each type of exercise has a specific way of adaptation, such as heart rate, time under tension, or maximum strength, which highlights another limitation for studies comparing different exercises.

## 5 Conclusion

Twenty‐four sessions of progressive intensity strength training did not provide a greater reduction in the fibromyalgia impact than constant intensity or walking exercises. Walking or strength training improves sleep quality, anxiety, depression, wind‐up mechanism, conditioned pain modulation, cutaneous sensory threshold, musculoskeletal performance, walking ability, and perceived improvement in people with fibromyalgia. However, the benefits are not sufficient to achieve a minimal clinically important difference, and patients do not adhere to treatment after 3 months without exercise.

## Author Contributions

**André Pontes‐Silva:** conceptualization, data curation, investigation, statistical analysis, methodology, validation, visualization, writing – original draft, writing – review and editing. **Almir Vieira Dibai‐Filho:** methodology, validation, visualization, writing – original draft, writing – review and editing. **Thayná Soares de Melo:** methodology, validation, visualization, writing – original draft, writing – review and editing. **Leticia Menegalli‐Santos:** methodology, validation, visualization, writing – original draft, writing – review and editing. **Josimari Melo DeSantana:** methodology, validation, visualization, writing – original draft, writing – review and editing. **Marcelo Cardoso de Souza:** methodology, validation, visualization, writing – original draft, writing – review and editing. **Mariana Arias Avila:** conceptualization, methodology, validation, visualization, writing – original draft, writing – review and editing.

## Funding

André Pontes‐Silva was funded by the FAPESP (grant 2022/08646‐6). This study was partially supported by the CAPES (finance code 001) and MCTI/FINEP (Proc. 01.19.0186.00). The funding source had no role in the study design, collection, analysis, interpretation of data, writing of the report, or in the decision to submit the article for publication.

## Ethics Statement

This study was approved by the Research Ethics Committee of the Universidade Federal de São Carlos (report number: 5.499.078).

## Consent

Informed consent was obtained from all subjects and/or their legal guardian(s). All respondents participated in this study freely and with their consent. All experiments were conducted in accordance with the tenets of the Declaration of Helsinki.

## Conflicts of Interest

André Pontes‐Silva serves as a reviewer for the *Musculoskeletal Care* and *Wiley Group*. The other authors declare no further competing interests.

## Data Availability

The data that support the findings of this study are available on request from the corresponding author. The data are not publicly available due to privacy or ethical restrictions.
